# Intravenous immunoglobulin treatment in a patient with adrenomyeloneuropathy

**DOI:** 10.1186/1471-2377-12-108

**Published:** 2012-09-26

**Authors:** Aia Elise Jønch, Else Rubæk Danielsen, Carsten Thomsen, Per Meden, Kirsten Svenstrup, Jørgen Erik Nielsen

**Affiliations:** 1 Kennedy Centre, Copenhagen University Hospital, Rigshospitalet, Gl. Landevej 7, Copenhagen, 2600, Glostrup, Denmark; 2 Department of Radiology, Copenhagen University Hospital, Rigshospitalet, Copenhagen, Denmark; 3 Department of Neurology, Copenhagen University Hospital, Bispebjerg Hospital, Copenhagen, Denmark; 4 Neurogenetics Clinic, Memory Disorders Research Unit, Department of Neurology, Copenhagen University Hospital, Rigshospitalet, Copenhagen, Denmark; 5 Section of Neurogenetics, Department of Cellular and Molecular Medicine, Faculty of Health Sciences, University of Copenhagen, Copenhagen, Denmark

**Keywords:** Adrenomyeloneuropathy, Limb pain, IVIG treatment, Magnetic resonance spectroscopy

## Abstract

**Background:**

Adrenomyeloneuropathy (AMN) is one of several phenotypes of the adrenoleukodystrophy spectrum caused by mutations in the *ABCD1* gene on the X chromosome. An inflammatory component is part of the disease complex ranging from severe childhood CNS demyelination to spinal cord and peripheral nerve degeneration.

**Case presentation:**

We present a patient with clinical progressive AMN and severe lower limb pain. Longitudinal brain magnetic resonance spectroscopy showed a constant slightly elevated myoinositol/total creatine ratio during the five year treatment period, probably reflecting demyelination, microglial activation and gliosis, indicating an inflammatory response. The pain was refractory to conventional therapy but intravenous immunoglobulin (IVIG) treatment was highly efficient.

**Conclusion:**

IVIG may be considered as a last resort for treatment of refractory pain in AMN patients with indications of an inflammatory component.

## Background

Adrenomyeloneuropathy (AMN) is the adult form of the progressive neurodegenerative condition, X-linked adrenoleukodystrophy. Mutations in the *ABCD1* gene result in a disrupted peroxisomal beta oxidation causing accumulation of the saturated very long chain fatty acids. The AMN phenotype is found in approximately 45% of the male patients with *ABCD1* mutations [1]. AMN presents in adult age with slowly progressive spastic paraplegia, sphincter disturbances, sensory disturbances, ataxia, pain and impotence [2]. AMN patients with the cerebral phenotype develop a cerebral inflammatory involvement with a faster progression, whereas patients with “pure” AMN can present with a mild inflammatory response mainly limited to the spinal cord and peripheral nerves [2,3].

We describe a patient with AMN who was treated successfully for severe leg pain with intravenous immunoglobulin (IVIG). During five years of treatment the disease progressed; however, magnetic resonance spectroscopy (MRS) revealed a stable, mildly elevated myoinositol (mI)/total creatine (Cr) ratio.

## Case presentation

A 48 year old Caucasian man was referred because of progressing pain, numbness, and stiffness of his lower limbs during 3-4 years. Lower urinary tract symptoms were absent. Neurological examination revealed hyperactive tendon reflexes in both arms and legs and bilateral Babinski reflexes. Except from a slightly increased tone distally in his legs he had an overall normal muscle tone and no limb weaknesses. The only abnormal sensory finding was an impaired sense of vibration in his first toes. He developed erectile dysfunction and reduced libido at 49 years of age. It was treated with sildenafil, tadalafil and later testosteronundecanoat with almost no effect.

Neurological examination at 55 years of age revealed a moderate lower limb spastic paraplegia, hyperactive deep tendon reflexes in both arms and legs, Babinski’s sign bilaterally, distally impaired thermal sensation and decreased sense of vibration distally of the knees, confirming clinical progression during the seven years.

The following investigations were normal: Routine blood biochemistry, including vitamin B12 concentration, methylmalonic acid, glycosylated hemoglobin, HTLV1 and HTLV2 (Human T-cell Lymphotropic Viruses), p-ANCA and c-ANCA (Anti-Neutrophil Cytoplasmic Antibodies), ANA (Anti-Nuclear Antibodies), HIV, syphilis, and arylsulfatase A activity. Cerebrospinal fluid analysis showed an increased spinal protein level of 0.78 g/l (normal 0.15-0.50 g/l), absent oligoclonal bands, and no Borrelia Burgdorferi antibodies.

Electrophysiological studies including EMG (electromyography), ENG (electroneurography), SSEP (somatosensory evoked potentials), and VEP (visual evoked potentials) were normal. MEP (motor evoked potentials) showed increased central conduction time (CCT) to muscles in the upper and lower extremities. The muscles investigated were biceps brachii (BB), flexor carpi radial is (FCR), first dorsal interosseus (FDI), tibialis anterior (TA), and abductor hallucis (AH). The CCT was increased by 45% and 34% to the right and left FCR, respectively. The CCT to the right side FDI was increased by 22% and the left side was normal. The CCT to TA was increased by 25% and 56% and to AH by 26% and 30% to right and left side, respectively. The CCT to both BB was normal. Peripheral conduction time was increased by 20% and 23% to right and left TA, respectively, and also to the right AH with 18%. The peripheral conduction times to BB, FCR and FDI were all normal.

The profile of saturated very long chain fatty acid (VLCFA) of serum was diagnostic of AMN, showing a ratio between C24 and C22 of 146% (normal 40-105%) and a ratio between C26 and C22 of 6.0% (normal 0-3%).

The AMN diagnosis was confirmed by identification of a novel c.2005C>T mutation in the *ABCD1* gene (Reference sequence: NM00033.3) predicting an amino acid shift from histidine to tyrosine at position 669 p. His669Tyr.

An ACTH stimulation test showed no adrenal insufficiency, but testosterone was slightly decreased (9.2 to 13 nmol/l; normal 10.0-28 nmol/l).

**Figure 1:**
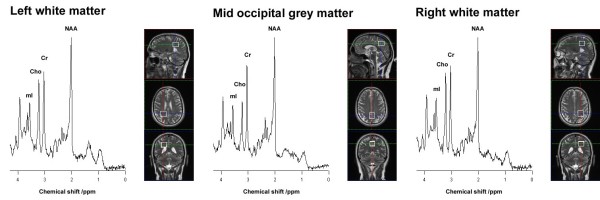
**Brain Magnetic Resonance Spectroscopy.** Short echo time magnetic resonance spectra acquired at three Tesla from left and right occipito-parietal primarily white matter and from mid-occipital primarily grey matter in the patient at age 52 years.

Repeated MRI’s (magnetic resonance imaging) of the brain and spinal cord during five years were normal. Short echo time MRS (magnetic resonance spectroscopy) studies were performed at ages 49, 50, 51, 53, and 55 years. The ratios of N-acetylaspartate (NAA), total choline (Cho), and mI to total Cr were calculated and results deviating more than 2 standard deviations (SD) from normal were regarded as abnormal. MRS showed elevated mI/Cr. The mI/Cr ratios were stable over time and on average 27% increased compared to the normal controls during the six years of study. The NAA/Cr ratios were low in the normal range or decreased to just below the normal range, but the NAA/Cr ratios did not change significantly over time. The Cho/Cr ratio stayed in the normal range all six years of study (Figure 1).

The patient had increasing pain from his legs during the years. The pain was described as initially a pricking and tingling sensation but later a constant, night and day, burning, stabbing pain, which was not precipitated by contact, temperature or movements.

The pain in his lower limbs was initially treated with peripheral analgesics, which had no effect. Later tizanidin, levodopa, amitriptylin, oxacarbazepin, and pregabalin were tried but all resulting in unacceptable side effects with no benefit.

Based on the involvement of an immune/inflammatory process in the pathogenesis of ALD/AMN, the increased CSF protein and the MRS data, immunosuppressive therapy with IVIG was initiated as a last resort. Treatment with IVIG was initialized in an in-house standard scheme of 30 g IVIG a day for three consecutive days every third month when the patient was 49 years old. Trying to increase the interval between treatments was unsuccessful because of worsening of the pain. The treatment was continued during five years approximately every third month and was administered when the symptoms deteriorated with increasing pain. The treatment led to clear improvement, reducing his pain from 9 to 3 on a “visual analogue scale” of 10.

## Discussion

Here we report on an AMN patient with a progressive spastic paraplegia, erectile dysfunction and severe pain in the lower limbs refractory to conventional treatment. A small fiber neuropathy may be the underlying cause of the pain but unfortunately the electrophysiological investigations aimed at revealing large fiber neuropathy and the patient was reluctant to participate in further electrophysiological investigations, skin biopsy or sural nerve biopsy. MRI of the brain was normal; however, cerebral involvement was revealed by MRS showing elevated mI/Cr. This finding probably reflects demyelination, microglial activation and gliosis, indicating a possible inflammatory component.

Spectroscopic evidence of biochemical abnormalities in AMN has previously been reported. Reduction of both NAA/Cho and NAA/Cr were found most severely in the internal capsule and parieto-occipital white matter, whereas no significant elevation of Cho/Cr was found. These results were suggested to reflect axonal damage in the brains of pure AMN patients [4]; however, the long echo time used in the study by Dubey and colleagues (2005), did not allow detection of mI and consequently a possible inflammatory component. Twenty percent of the “pure” AMN patients develop the cerebral phenotype [3] and based on the MRS we suggest classifying our patient as an inflammatory cerebral type AMN.

Currently, the most efficient therapeutic opportunity for patients with the cerebral form of adrenoleukodystrophy (ALD) is hematopoietic stem cell transplantation, whereas applications of immunomodulatory and immunosuppressive drugs have failed to prevent progression of cerebral neuroinflammation [5].

Heterogeneous peripheral and central mechanisms are presumably involved in the pain related to AMN. The exact pathophysiological mechanisms are unknown, but might depend of antibodies to neuronal structures as seen in neuropathy associated with diabetes [6]. Furthermore, cytokines and growth factors have been suggested to play a role in initiating or maintaining neuropathic pain [7]. IVIGs have different actions, including modulation of pathogenic autoantibodies and inhibition of complement activation and cytokines [8], which might be related to the beneficial effect in our patient. IVIG was previously reported to reduce neuropathic pain associated with Sjögren's syndrome, an autoimmune disease, and to reduce pain and improve motor function markedly in proximal diabetic neuropathy [9,10].

IVIG treatment has hitherto not been reported in AMN but previously administered to ALD patients without clear effect on disease progression and it is presently not a recommended treatment [5]. MRS revealed normal Cho/Cr and the NAA/Cr ratios in our patient and those ratios did not change significantly during the follow up period. IVIG was highly effective in the pain treatment, but whether the IVIG treatment influenced the disease course, prevented axonal damage in the brain and stabilized the mI/Cr remains speculative. The present study should be interpreted with caution and IVIG is not recommended for AMN in general; however, as a last resort for treating otherwise refractory pain in AMN, IVIG may be explored.

## Conclusion

The patient with a novel c.2005C>T mutation in the *ABCD1* gene had an AMN phenotype with severe leg pain. According to MRS, indicating a coexisting cerebral demyelination and inflammation he was classified as AMN cerebral phenotype. IVIG treatment relieved the severe pain and it may be considered in otherwise treatment-refractory pain of AMN, cerebral type.

## Consent

Written informed consent was obtained from the patient for publication of this Case report and any accompanying images. A copy of the written consent is available for review by the Series Editor of this journal.

## Competing interests

The authors declare to have no competing interests.

## Authors’ contributions

AEJ: Study concept and design, analysis and interpretation of the data, acquisition of the data, drafting and revising the manuscript. ERD, CT: Analysis and interpretation of the MR data, acquisition of the data, drafting and revising the manuscript. KS, PM: Analysis and interpretation of the clinical data, acquisition of the data, drafting and revising the manuscript. JEN: Study concept and design, analysis and interpretation of the data, drafting/revising the manuscript, study supervision. All authors have read and approved the final manuscript.

## Pre-publication history

The pre-publication history for this paper can be accessed here:

http://www.biomedcentral.com/1471-2377/12/108/prepub
